# The Building of a Brain

**Published:** 1874-11

**Authors:** 


*The Building of a Brain.* By EDWARD H. CLARKE, M. D. Boston; James R. Osgood & Co., 1874.

Dr. Clarke's "Sex in Education," has made him a wide acquaintance among educators, both in this country and abroad. The attention which that little book attracted, and the many answers and criticisms which it elicited, both favorable and unfavorable, showed that the subject was one upon which a little well-digested information would do no harm.

The present book is the outgrowth of an essay read before the National Educational Association, which met in Detroit.

The book is divided into three parts: Nature's Working Plans, An Error in Female-building, and A Glimpse at English Brain-building, of which part first constitutes the address delivered before the association.

These essays are a further exposition of Dr. Clarke's ideas as expressed in "Sex in Education."

The first essay is an argument in favor of a general and systematic developement of body and mind in unison, an appeal against the plan of teaching so generally pursued, of making a scholar learn so much from a given text book in a given time, regardless of physical or mental conditions, and often, we would add, regardless of whether the subject is at all understood by the pupil. It seems to be to often the plan to compel a scholar to commit to memory so much printed matter, with no regard as to whether the subject is understood or not, the teacher being satisfied if the pupil can repeat the words of the author *verbatim.*

It seems undoubtedly true that too little attention is paid to the requirements of the body by those who attempt to develope the brain, and in these times, when the agitators of so-called woman's rights are clamoring for the admission of women into all the various pursuits followed by men, some one is needed to point out to them the danger of such a course. We would not deny to women the opportunity of obtaining the highest mental culture possible, but when that culture is to be obtained at the expense of physical health and the sacrifice of those traits which are so distinctly feminine, we ask, is the prize worth the cost? If girls are to be educated to as high a degree as boys, and why should they not be? it must not be in the same manner. Their physical natures are widely different, and teachers and parents should have the importance of this fact fully impressed upon them. Dr. Clarke's writings have attracted a large amount of attention, and we hope done some good. Those who have read "Sex in Education," should also read this later essay.

